# Bright kidney: a sign of contrast induced nephropathy after percutaneous coronary intervention

**DOI:** 10.1093/omcr/omaf110

**Published:** 2025-07-27

**Authors:** Kazunori Omote, Tadao Aikawa, Daisuke Sunaga, Naohiro Funayama

**Affiliations:** Department of Cardiology, Hokkaido Cardiovascular Hospital, Minami-27 Nishi-13, Chuo-ku, Sapporo, Hokkaido 064-8622, Japan; Department of Cardiology, Hokkaido Cardiovascular Hospital, Minami-27 Nishi-13, Chuo-ku, Sapporo, Hokkaido 064-8622, Japan; Department of Cardiovascular Biology and Medicine, Juntendo University Graduate School of Medicine, 2-1-1 Hongo, Bunkyo-ku, Tokyo 113-8421, Japan; Department of Cardiology, Hokkaido Cardiovascular Hospital, Minami-27 Nishi-13, Chuo-ku, Sapporo, Hokkaido 064-8622, Japan; Department of Cardiology, Hokkaido Cardiovascular Hospital, Minami-27 Nishi-13, Chuo-ku, Sapporo, Hokkaido 064-8622, Japan

**Keywords:** contrast induced nephropathy, non-contrast computed tomography

An 85-year-old woman was referred to our hospital due to chest pain. Based on the results of blood tests and electrocardiography, she was diagnosed with acute myocardial infarction, prompting urgent percutaneous coronary intervention (PCI). The patient’s baseline serum creatinine was 1.21 mg/dl (estimated glomerular filtration rate: 33 ml/min/1.73m^2^). A total of 240 ml of iodinated contrast (Iohexol) was administered during PCI. Echocardiography revealed mildly reduced left ventricular ejection fraction of 44%, with no significant wall motion abnormalities aside from the infarcted region. The day after the PCI, her serum creatinine level increased, and her urine output decreased. The patient was treated with intravenous fluids and diuretics; however, her renal function continued to deteriorate, eventually reaching anuric state. To differentiate post-renal causes of acute kidney injury from contrast-induced nephropathy (CIN), non-contrast computed tomography (CT) was performed 4 days after the PCI. Unexpectedly, the kidney parenchyma exhibited significant enhancement without urinary tract obstruction, which was consistent with CIN (Figs 1 and 2).

**Figure 1:**
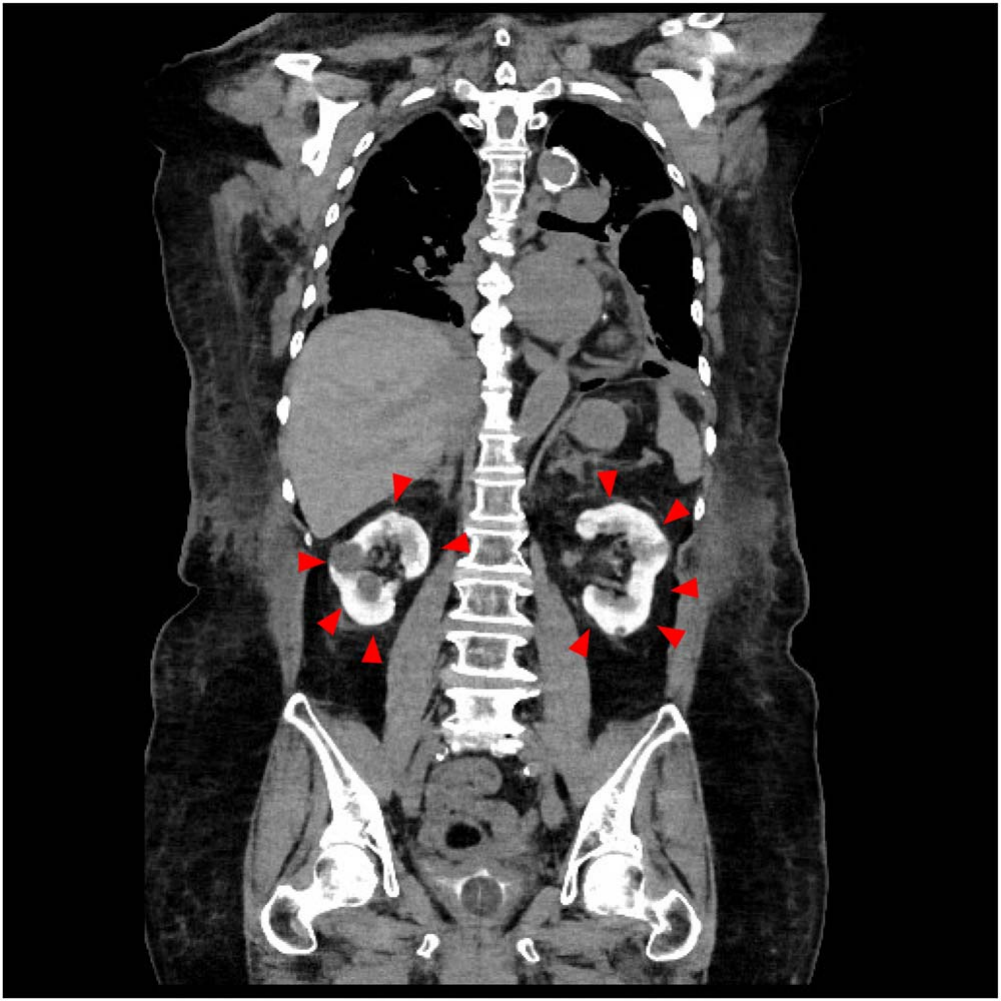
Bright kidney detected by non-contrast computed tomography 4 days after the percutaneous coronary intervention (axial).

**Figure 2:**
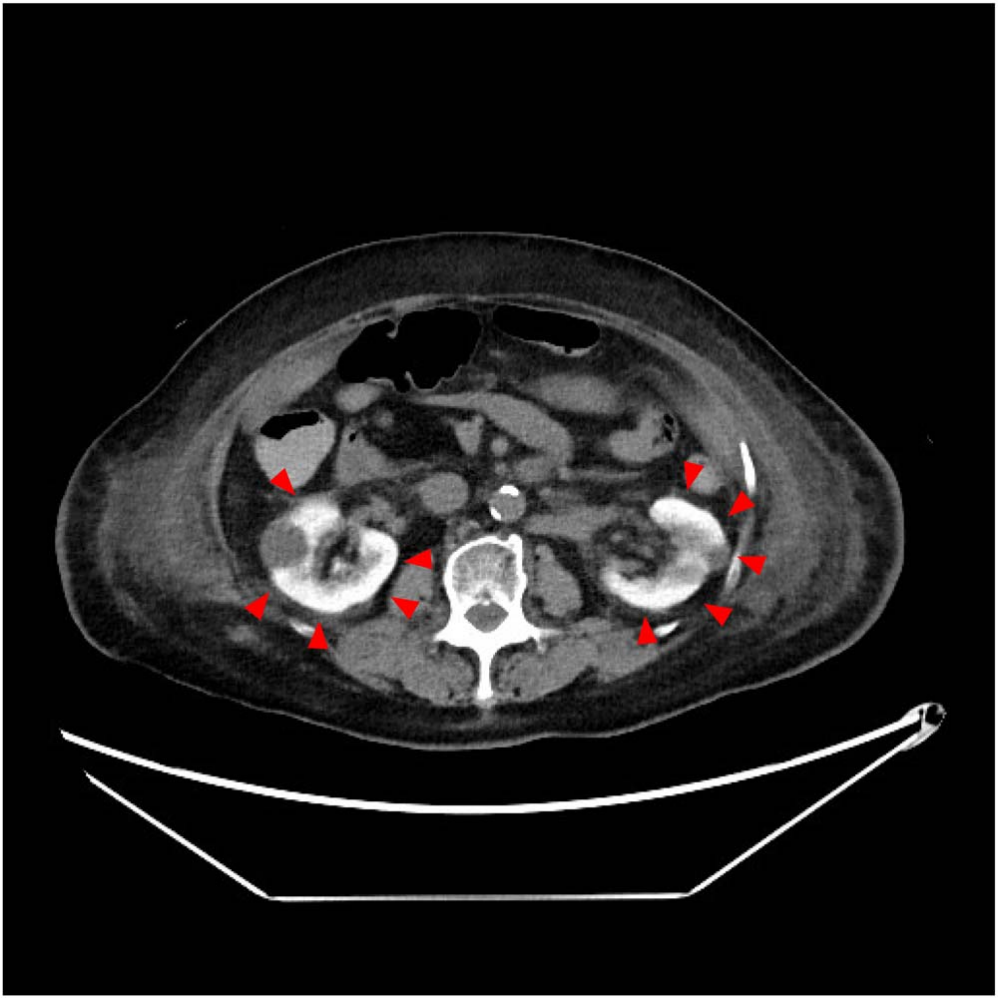
Bright kidney detected by non-contrast computed tomography 4 days after the percutaneous coronary intervention (coronal).

## Discussion

In patients with normal renal function, contrast retention in the kidneys usually resolves within 24 hours [1]. However, in cases of CIN, impaired glomerular filtration may lead to prolonged retention. Not all cases of CIN will demonstrate persistent contrast retention visible on non-contrast CT. The degree of enhancement may depend on the severity of renal impairment, timing of imaging, and the type and amount of contrast used. This case underscores the utility of the ‘bright kidney’ sign on non-contrast CT several days post-PCI as a practical and underrecognized radiologic indicator of CIN in the acute setting. CIN after PCI is associated with adverse cardiac events and increased short- and long-term mortality [2]. The present case suggests that ‘bright kidney,’ defined as sustained contrast-filling of the kidneys on non-contrast CT following PCI, may serve as a potential indicator of CIN.
